# Predicting clinical events characterizing the progression of amyotrophic lateral sclerosis via machine learning approaches using routine visits data: a feasibility study

**DOI:** 10.1186/s12911-024-02719-5

**Published:** 2024-10-29

**Authors:** Alessandro Guazzo, Michele Atzeni, Elena Idi, Isotta Trescato, Erica Tavazzi, Enrico Longato, Umberto Manera, Adriano Chió, Marta Gromicho, Inês Alves, Mamede de Carvalho, Martina Vettoretti, Barbara Di Camillo

**Affiliations:** 1 Department of Information Engineering, University of Padova, Padua, Italy; 2 Department of Neurosciences Rita Levi Montalcini, University of Turin, Turin, Italy; 3 Faculdade de Medicina, IMM J. L. Antunes, Universidade de Lisboa, Lisbon, Portugal; 4 Department of Comparative Biomedicine and Food Science, University of Padova, Padua, Italy

**Keywords:** Amyotrophic lateral sclerosis, Multi-layer perceptron, Logistic regression, Explainability

## Abstract

**Background:**

Amyotrophic lateral sclerosis (ALS) is a progressive neurodegenerative disease that results in death within a short time span (3-5 years). One of the major challenges in treating ALS is its highly heterogeneous disease progression and the lack of effective prognostic tools to forecast it. The main aim of this study was, then, to test the feasibility of predicting relevant clinical outcomes that characterize the progression of ALS with a two-year prediction horizon via artificial intelligence techniques using routine visits data.

**Methods:**

Three classification problems were considered: predicting death (binary problem), predicting death or percutaneous endoscopic gastrostomy (PEG) (multiclass problem), and predicting death or non-invasive ventilation (NIV) (multiclass problem). Two supervised learning models, a logistic regression (LR) and a deep learning multilayer perceptron (MLP), were trained ensuring technical robustness and reproducibility. Moreover, to provide insights into model explainability and result interpretability, model coefficients for LR and Shapley values for both LR and MLP were considered to characterize the relationship between each variable and the outcome.

**Results:**

On the one hand, predicting death was successful as both models yielded F1 scores and accuracy well above 0.7. The model explainability analysis performed for this outcome allowed for the understanding of how different methodological approaches consider the input variables when performing the prediction. On the other hand, predicting death alongside PEG or NIV proved to be much more challenging (F1 scores and accuracy in the 0.4-0.6 interval).

**Conclusions:**

In conclusion, predicting death due to ALS proved to be feasible. However, predicting PEG or NIV in a multiclass fashion proved to be unfeasible with these data, regardless of the complexity of the methodological approach. The observed results suggest a potential ceiling on the amount of information extractable from the database, e.g., due to the intrinsic difficulty of the prediction tasks at hand, or to the absence of crucial predictors that are, however, not currently collected during routine practice.

## Introduction

Amyotrophic lateral sclerosis (ALS) is a rapidly progressive neurodegenerative disease that affects motor neurons in the brain and spinal cord, leading to muscle weakness, atrophy, and, ultimately, paralysis. The disease typically results in death within a relatively short span of 3-5 years, although survival times can vary widely and depend on many factors including age, site of onset, rate of disease progression, and presence of comorbidities [1]. ALS is also characterized by significant heterogeneity in its progression across the patient population, with some individuals showing a slow progression and others experiencing a rapid decline [2].

One of the major challenges in treating ALS is the lack of effective prognostic tools for predicting disease progression. Precise prognostic tools would facilitate improved drug development through more cost-effective and accurate clinical trials, while also offering valuable insights into disease progression. In recent years, artificial intelligence (AI) approaches have shown promise for predicting clinical outcomes in various disease contexts, including ALS [3–6]. By leveraging complex algorithms and large amounts of data, AI-based models can identify patterns and relationships that may not be immediately apparent to human observers. In the case of ALS, this could potentially lead to more accurate prognostic tools that could help clinicians tailor treatment plans to individual patients. Several ALS clinical outcomes can be predicted via AI models, such as the evolution of ALS functional rating scale ALSFRS or its revised version ALSFRS-R [7, 8], the forced vital capacity (FVC) value [9], and the occurrence of relevant events related to disease progression, i.e., the need for non-invasive ventilation (NIV) [10], tracheostomy [11], or death [12, 13]. Most literature studies approach the prediction problem using classification modeling techniques (e.g., random forests or logistic regression (LR)), few studies opt for survival analysis approaches such as the Cox model, and only a handful of studies employ deep-learning techniques. Overall, the literature concerning the development of predictive models for clinical outcomes of ALS is still limited as most studies focus on the identification of risk factors based on statistical analyses rather than providing a prediction model [14]. Another relevant factor influencing the limited model development is the disease’s rarity [15] which makes collecting enough high-quality data quite challenging. Hence, most studies approach the problem using small datasets obtained from specific clinical trials [16]. These approaches achieve good predictive power, with an Area Under the Receiver-Operating Curve (AUROC) $> 0.8$. However, as the considered variables are trial-specific and thus not necessarily collected during everyday clinical practice, their translation into real-world applications is not easily performed.

In a previous study from our group [6], the problem of predicting relevant clinical events for ALS was considered within a survival analysis framework. To follow up on our previous work and provide a complementary perspective, in this study, the objective was to explore the possibility of using AI-based approaches in a single and multiclass classification framework to predict relevant clinical outcomes that characterize the progression of ALS via readily available data collected during routine visits.

Two types of AI models (LR and multilayer perceptron (MLP)) were developed and tested to predict three clinical outcomes of great interest to clinicians: death, percutaneous endoscopic gastrostomy (PEG), and non-invasive ventilation (NIV) within two years from the first visit. A first binary classification problem was defined based on the event of death. A second three-class problem was defined distinguishing instances where no events occur within two years (class 0), instances where death occurs within 2 years and before PEG (class 1), and instances where PEG is performed within 2 years and before death (class 2). Finally, a third classification problem was defined similarly to the second one, but considering NIV instead of PEG as a clinical event of interest. Moreover, this feasibility analysis is completed by providing insights into model explainability. Specifically, the strength of the relation between each variable and the outcome was evaluated by considering the estimated model coefficients for LR and the SHapley Additive exPlanations (SHAP) values [17] for both LR and MLP. Results suggested that while the considered AI approaches had acceptable predictive performance for the binary classification task of predicting death within two years, combining the prediction of death with either PEG or NIV in a multiclass classification problem is more challenging considering the available data. Our study provides valuable insights into the potential and limitations of AI-based predictive models for ALS using input variables obtained from routine visits.

## Data and methods

### Dataset and preprocessing

The dataset used in this study was provided by the European Horizon 2020 project *Bringing artificial intelligence home for a better care of amyotrophic lateral sclerosis and multiple sclerosis* (BRAINTEASER) [18]. The BRAINTEASER project aims to use AI to gain a better understanding of ALS, predict disease progression, and propose interventions to delay its advancement. This involves developing models that can identify and forecast disease outcomes over time for different patient groups, providing support for patient care and clinical trials. Detecting complications during the disease progression is crucial for ALS patients and healthcare professionals.

**Table 1:** List of variables considered as inputs for the models. Baseline variables were obtained from raw data collected at the first visit meanwhile follow-up variables were obtained from raw data collected at multiple visits within 6 months after the index date

Section	Sub-section	Variables
**Baseline**	Demographics	age_onset, sex, job_qualification
	Anthropometrics	bmi_premorbid, bmi_baseline, slope_weight
	ALS onset and diagnosis	onset_bulbar, onset_limb_lower, onset_limb_upper, time_since_onset, diagnostic_delay
	ALSFRS-R subscores	alsfrsr_baseline_breathing, alsfrsr_baseline_bulbar, alsfrsr_baseline_lower_limbs, alsfrsr_baseline_trunk, alsfrsr_baseline_upper_limbs
	Genetic mutations	C9orf72_mutation, SOD1_mutation
	Previous pathologies	autoimmune_disease, stroke, thyroid_disorder, hypertension, primary_neoplasm, ALS_familiar_history
	Lifestyle	smoking
**6 months follow-up**	ALSFRS-R progression slopes	alsfrsr_slope_progression_breathing, alsfrsr_slope_progression_bulbar,
		alsfrsr_slope_progression_lower_limbs, alsfrsr_slope_progression_trunk,
		alsfrsr_slope_progression_upper_limbs
	ALSFRS-R min and max values	alsfrsr_max_breathing, alsfrsr_max_bulbar,
		alsfrsr_max_lower_limbs, alsfrsr_max_trunk,
		alsfrsr_max_upper_limbs, alsfrsr_min_breathing,
		alsfrsr_min_bulbar, alsfrsr_min_lower_limbs,
		alsfrsr_min_trunk, alsfrsr_min_upper_limbs
	Test	fvc

The ALS dataset, provided within the BRAINTEASER project, includes data coming from two data registries, one Italian and one Portuguese. On one hand, the Italian ALS data registry is based on the Piemonte and Valle d’Aosta Register for Amyotrophic Lateral Sclerosis (PARALS) [19]. PARALS is an epidemiologic prospective register that covers two Italian regions (population of 4,476,931 inhabitants according to the 2011 census). Demographic and clinical data from 3,257 ALS patients collected from January 1, 1995, through December 31, 2018 were considered from this registry. On the other hand, the Lisbon ALS registry contains demographic and clinical data from 1,562 ALS patients regularly followed at the ALS clinic at Hospital de Santa Maria, Lisbon since 1995 and last updated in October 2021. The two registries were harmonized to obtain a set of common variables to be used as model inputs. The list of input variables is reported in Table 1. age_onset is the patient’s age evaluated at the onset of the disease, sex is a binary variable equal to 0 if the patient is male and 1 if female, job_qualification is a categorical variable with four values representing four levels of qualification required for a job, bmi_premorbid is the patient’s BMI evaluated before the onset of the disease, bmi_baseline is the patient’s BMI evaluated at the baseline, slope_weight is the weight rate of change evaluated during the 6 months follow-up. onset_bulbar, onset_limb_lower, and onset_limb_upper are binary variables describing the ALS onse type [20]. time_since_onset is the time difference between the baseline and the onset while diagnostic_delay is the time difference between the diagnosis and the baseline. Typically, the onset is before the first visit and the diagnosis is after, both these times are positive for the majority of patients. alsfrsr_baseline_* variables represent the ALSFRS-R subscores evaluated at the baseline for the breathing, bulbar, trunk, lower and upper limbs domains [21]. C9orf72_mutation and SOD1_mutation are two binary variables equal to 1 if the corresponding genetic mutation was observed in the patient. autoimmune_disease, stroke, thyroid_disorder, hypertension, primary_neoplasm, and ALS_familiar_history are binary variables equal to 1 if the pathology was observed for the patient. Smoking is a binary variable equal to 1 if the patient is a smoker. alsfrsr_slope_progression_* variables represent the ALSFRS-R subscores rate of change evaluated during the 6-month follow-up for the breathing, bulbar, trunk, lower and upper limbs domains. alsfrsr_max_* and alsfrsr_min_* variables represent the maximum and minimum ALSFRS-R values observed during the 6-month follow-up. fvc is a variable reporting the last FVC measurement of the patient. Summary statistics of each variable are reported in Table 2 in “Population characteristics” section.

The input data were processed by following the procedure described in [6]. The key points of such data processing were:

- Map categorical variables with two levels to Boolean variables, and convert multinomial variables to dummy variables.
- Compute the rate of change during the observation period, together with the minimum and maximum observed values, to account for dynamic changes in the data.
- Exclude variables with more than 70% of missing values or with <1% subjects having a value different from the majority.
- Normalization via min-max scaling.
- Imputation of missing values using multivariate imputation by chained equations [22] carefully considering the parameters of the imputation method and performing a sensitivity analysis to mitigate risks related to the imputation of sensitive data.

The exclusion criteria for the subjects were:

- More than 20% of the variables with a missing value.
- Absence of ALSFRS-R measurements, as this was considered a fundamental piece of information for ALS patients, accounting for and clearly explicating the health status of the subjects.
- Inconsistent clinical history, e.g., subjects for whom there were events recorded after the death date.

Outcome variables were processed to consider the problem from a classification perspective as an alternative to the survival analysis perspective already explored in [6, 23]. Consequently, the overall dataset was used to derive three sub-datasets, one for each outcome of interest (death, death or PEG, and death or NIV). For the first sub-dataset (*N* = 2100 patients), a binary outcome was considered, specifically, the label 1 was assigned to patients who died within two years after the first visit (1058 out of 2100, 50%), meanwhile, the label 0 was assigned to those that survived (1042 out of 2100, 50%). The second sub-dataset (*N* = 2027 patients) considered two possible events, namely death or PEG, always occurring within two years from the first visit. In this case, the label 1 was assigned to patients for which the first recorded outcome was death (684 out of 2027, 33.7%), the label 2 was assigned to patients for which the first recorded outcome was PEG (455 out of 2027, 22.4%), and the label 0 was assigned to patients that experienced neither of the two events within the two years (888 out of 2027, 43.8%). Finally, also the third sub-dataset (*N* = 1739 patients) considered two possible events, namely death or NIV, occurring within two years from the first visit. In this case, the label 1 was assigned to patients for which the first recorded outcome was death (497 out of 1739, 28.5%), the label 2 was assigned to patients for which the first recorded outcome was NIV (589 out of 1739, 34%), and the label 0 was assigned to patients that experienced neither of the two events within the two years (653 out of 1742, 37.5%).

Each of the three sub-datasets was then divided into three subsets: training, validation, and test sets, with each set comprising 70%, 15%, and 15% of the total dataset, respectively. Thus, for the death outcome, 1470 patients were considered in the training set, 315 in the validation set, and 315 in the test set. For the PEG outcome, 1418 patients were considered in the training set, 305 in the validation set, and 304 in the test set. For the NIV outcome, 1217 patients were considered in the training set, 261 in the validation set, and 261 in the test set.

### AI model development and evaluation

Two supervised machine learning approaches were considered to solve the three classification problems, a simple linear approach, namely a LR with L2 regularization, and a more complex non-linear approach, namely a deep learning MLP. To ensure the technical robustness and reproducibility of the study a two-step optimization framework was implemented.

The first step performs hyperparameter optimization by the random search approach for both LR and MLP. The LR requires optimization of the regularization parameter C defining the L2 penalty term (uniformly sampled in the range [0.0001, 1.0] according to a logarithmic scale). The MLP requires optimization of various hyperparameters, such as learning rate (sampled from a descending sequence ranging in [0.01, 0.0001], with each number being one-tenth of the previous number), initializer (sampled from all the possible initializers, i.e., random normal, random uniform, glorot normal, he normal, he uniform, variance scaling, orthogonal), activation function (sampled from all the possible activation functions, i.e., relu, tanh, selu, elu), and architecture complexity (a funnel-like structure where the number of nodes in the next layer is half the number of nodes in the previous layer, with a maximum number of starter nodes ranging in [8-512]). For each random search step, a five-fold cross-validation was performed on the training set to obtain the mean cross-validation cross-entropy loss. The best hyperparameters set was chosen based on the minimum cross-validation cross-entropy loss obtained over 500 random iterations.

The second optimization step consisted of the training, on the whole training set, of several models using the optimal set of hyperparameters (obtained from the previous step) and different random initializations for the optimizer. The best model was chosen by minimizing the cross-entropy loss computed on the validation set among 100 random iterations.

Finally, the optimal model was tested on an independent portion of the data considering as performance evaluation metrics the Area under the Precision-Recall Curve (AUPRC) and the AUROC as well as precision, recall, F1 score, accuracy, and Matthews correlation coefficient (MCC) computed after thresholding. For binary classification, the threshold was set using the validation set by testing all the predicted probabilities and selecting the one that maximized the geometric mean between specificity and sensitivity. In the multiclass case, instead, the class was assigned by selecting the one with the highest predicted probability in a 1 vs. all fashion.

### AI model explainability

To provide insights into model explainability and understand which variables are the most influential towards the prediction the relationship of each feature with the outcome is studied. For the LR approach, such a relationship is evaluated through its estimated regression coefficient as well as through SHAP values. Instead, for the MLP approach, which lacks coefficients directly associated with the importance of each variable, only SHAP values are considered. For each variable of a given input sequence, SHAP values are computed by evaluating how prediction is affected when its value is permutated with those of other elements randomly sampled from other input sequences constituting the background set. In order to compute SHAP values, all input sequences belonging to the test set were independently interpreted using, for each one, 500 elements randomly sampled from the training set that was thus used as a background set [24]. Finally, the distribution of all SHAP values for a variable was depicted using violin plots [25]. As all available variables were used to train the models and no feature selection was performed, compensation effects due to the presence of collinear variables may appear in the results. Consequently, the explainability analysis is completed with a collinearity analysis performed by computing Pearson’s correlation coefficients [26] for all variables combinations. All correlation coefficients are displayed on a matrix via a color code where blue means positive correlation and red negative correlation.

## Results and discussion

### Population characteristics

Table 2 shows insights into the population characteristics of the three considered datasets. Continuous variables are presented with mean and standard deviation while binary variables as a percentage relative to N subjects (*N* = 2100 death outcome, *N* = 2027 death or PEG outcome, *N* = 1739 death or NIV outcome). Variables seem to be similarly distributed across the three considered datasets, this result was somewhat expected as a common dataset given by the merge of the Italian and Portuguese registries was considered to obtain three different sub-datasets characterized by different outcomes of interest. Overall, patients in this data were old ($\sim$65 years on average), female ($\sim$55%), with few previous pathologies and a mild impairment caused by ALS.

**Table 2:** Summary statistics of all considered variables of the three considered datasets. Continuous variables are presented with mean and (standard deviation), binary variables as a percentage relative to N subjects (*N* = 2100 death outcome, *N* = 2027 death or PEG outcome, *N* = 1739 death or NIV outcome)

	Outcomes		
Variable name	Death	Death or PEG	Death or NIV
**age_onset**	64.7 (11.3)	63.3 (10.5)	65.6 (12.2)
**male sex**	45.7 %	43.5 %	44.6 %
**job_qualification**	1 : 62.4 %	1 : 61.2 %	1 : 63.6 %
	2 : 12.0 %	2 : 13.1 %	2 : 11.7 %
	3 : 21.8 %	3 : 22.6 %	3 : 20.8 %
	4 : 3.8 %	4 : 3.1 %	4 : 3.9 %
**bmi_premorbid**	26.0 (4.1)	25.8 (4.0)	26.5 (4.3)
**bmi_baseline**	24.7 (4.1)	23.6 (4.2)	25.1 (3.9)
**slope_weight**	0.35 (0.68)	0.34 (0.67)	0.32 (0.69)
**onset_bulbar**	32.0%	31.1%	33.2%
**onset_limb_lower**	36.7%	37.3%	35.7%
**onset_limb_upper**	29.7%	28.9%	29.9%
**time_since_onset**	17.3 (13.3)	16.4 (12.2)	18.1 (14.1)
**diagnostic_delay**	11.6 (10.2)	12.4 (11.2)	12.4 (11.1)
**alsfrsr_baseline_breathing**	11.5 (1.2)	10.9 (1.4)	10.5 (1.1)
**alsfrsr_baseline_bulbar**	10.4 (2.0)	10.1 (1.6)	10.6 (2.2)
**alsfrsr_baseline_lower_limbs**	5.8 (2.2)	5.2 (2.0)	5.9 (2.5)
**alsfrsr_baseline_upper_limbs**	6.4 (1.8)	5.9 (1.7)	6.6 (1.7)
**alsfrsr_baseline_trunk**	6.4 (1.8)	6.1 (1.9)	6.0 (1.7)
**C9orf72_mutation**	4.7%	4.3%	4.9%
**SOD1_mutation**	1.4%	1.2%	1.6%
**autoimmune_disease**	2.0%	2.5%	1.9%
**stroke**	3.0%	2.7%	3.5%
**thyroid_disorder**	9.0%	9.6%	8.4%
**hypertension**	45.0%	43.4%	46.2%
**primary_neoplasm**	9.8%	8.8%	10.3%
**ALS_familiar_history**	8.0%	7.5%	9.2%
**smoking**	39.2%	38.1%	37.5%
**alsfrsr_slope_progression_breathing**	- 0.03 (0.17)	- 0.03 (0.15)	- 0.02 (0.21)
**alsfrsr_slope_progression_bulbar**	- 0.06 (0.22)	- 0.05 (0.18)	- 0.06 (0.20)
**alsfrsr_slope_progression_lower_limbs**	- 0.07 (0.24)	- 0.06 (0.31)	- 0.06 (0.19)
**alsfrsr_slope_progression_upper_limbs**	- 0.05 (0.19)	- 0.04 (0.18)	- 0.06 (0.22)
**alsfrsr_slope_progression_trunk**	- 0.05 (0.18)	- 0.05 (0.16)	- 0.06 (0.12)
**alsfrsr_max_breathing**	11.6 (1.1)	10.9 (1.3)	11.4 (1.5)
**alsfrsr_max_bulbar**	10.4 (2.0)	11.1 (2.3)	9.9 (1.8)
**alsfrsr_max_lower_limbs**	5.8 (2.2)	5.9 (2.5)	5.7 (2.3)
**alsfrsr_max_upper_limbs**	6.5 (1.7)	5.8 (1.1)	6.9 (1.9)
**alsfrsr_max_trunk**	6.5 (1.8)	6.9 (1.2)	5.7 (1.3)
**alsfrsr_min_breathing**	10.8 (1.9)	11.1 (2.3)	10.1 (1.3)
**alsfrsr_min_bulbar**	9.5 (2.8)	9.8 (2.2)	9.1 (2.1)
**alsfrsr_min_lower_limbs**	4.7 (2.5)	5.0 (2.1)	4.2 (2.3)
**alsfrsr_min_upper_limbs**	5.5 (2.2)	5.1 (2.7)	5.7 (1.8)
**alsfrsr_min_trunk**	5.4 (2.5)	5.2 (2.9)	5.9 (2.3)
**fvc**	82.7 (25.5)	81.0 (22.8)	85.6 (27.2)

### Death prediction

The performance metrics obtained when considering the death outcome are reported in Table 3. Both models (LR and MLP) performed well on the independent test set reaching F1 scores well above 0.7 with AUPRC and AUROC that were both $>0.8$. Overall, the two methods led to comparable results with the MLP achieving higher recall and LR performing better in terms of precision instead.

**Table 3:** Performance Evaluation of Death Prediction (*N* = 315): Results in the test set are reported using Area Under the Precision-Recall Curve (AUPRC), Area Under the Receiver Operating Characteristic Curve (AUROC), precision (P), recall (R), F1 score (F1), accuracy (Acc), and Matthews Correlation Coefficient (MCC). The cross-entropy loss (CV-L) expressed as mean ± standard deviation and test-set loss (TL) are reported as well

Model	AUPRC	AUROC	P	R	F1	Acc	MCC	CV-L	TL
LR	0.81	0.79	0.74	0.74	0.74	0.73	0.45	0.55±0.05	0.58
MLP	0.84	0.82	0.71	0.82	0.76	0.73	0.47	0.57±0.03	0.53

### Death or PEG prediction

The performance metrics obtained when considering the death or PEG outcome are reported in Table 4. Both the LR and MLP models showed promising predictive performance in the one-versus-all case for predicting the absence of an event (class 0) versus the occurrence of death or PEG (classes 1 or 2), reaching AUPRC and AUROC of $\sim 0.83$ and F1 score of 0.74 for both models. On the contrary, predicting death (class 1) vs. no event or PEG (classes 0 or 2) and PEG (class 2) vs. no event or death (classes 0 and 1) led to general lower predictive performance (AUPRC and F1 score $\sim 0.6$).

**Table 4:** Performance Evaluation of Death or PEG Prediction (*N* = 304): Results in the test set are reported using Area Under the Precision-Recall Curve (AUPRC), Area Under the Receiver Operating Characteristic Curve (AUROC), precision (P), recall (R), F1 score (F1), accuracy (Acc), and Matthews Correlation Coefficient (MCC). The cross-entropy loss (CV-L) expressed as mean ± standard deviation and test-set loss (TL) are reported as well

M	O	AUPRC	AUROC	P	R	F1	Acc	MCC	CV-L	TL
LR	No event	0.82	0.83	0.68	0.82	0.74	-	-	-	
	Death	0.60	0.76	0.59	0.53	0.56	-	-	-	
	PEG	0.68	0.86	0.78	0.55	0.64	-	-	-	
							0.67	0.45	0.88±0.05	0.76
MLP	No event	0.81	0.86	0.71	0.77	0.74	-	-	-	
	Death	0.62	0.75	0.6	0.5	0.55	-	-	-	
	PEG	0.5	0.8	0.51	0.55	0.53	-	-	-	
							0.62	0.45	0.87±0.04	0.79

### Death or NIV prediction

The performance metrics obtained when considering the death or NIV outcome are reported in Table 5. Both LR and MLP achieved their best performance when predicting the absence of adverse events and instead struggled when distinguishing between death and NIV. Interestingly, the prediction of NIV seems to be a more challenging task whose difficulties can be related to the high variability in the timing of this intervention.

**Table 5:** Performance Evaluation of Death or NIV Prediction (*N *= 261): Results in the test set are reported using Area Under the Precision-Recall Curve (AUPRC), Area Under the Receiver Operating Characteristic Curve (AUROC), precision (P), recall (R), F1 score (F1), accuracy (Acc), and Matthews Correlation Coefficient (MCC). The cross-entropy loss (CV-L) expressed as mean ± standard deviation and test-set loss (TL) are reported as well

M	O	AUPRC	AUROC	P	R	F1	Acc	MCC	CV-L	TL
LR	No event	0.60	0.76	0.49	0.76	0.60	-	-	-	
	Death	0.65	0.75	0.58	0.48	0.53	-	-	-	
	NIV	0.55	0.69	0.55	0.36	0.43	-	-	-	
							0.53	0.35	0.95±0.04	0.94
MLP	No event	0.72	0.80	0.57	0.72	0.64	-	-	-	
	Death	0.56	0.74	0.55	0.39	0.45	-	-	-	
	NIV	0.58	0.69	0.51	0.48	0.49	-	-	-	
							0.54	0.31	0.96±0.04	0.96

### Model explainability results

Here, model explainability analysis is fully discussed only for the death outcome as this was the outcome for which the developed models led to reliable predictive performance thus leading to reliable explainability insights as well.

**Fig. 1:**
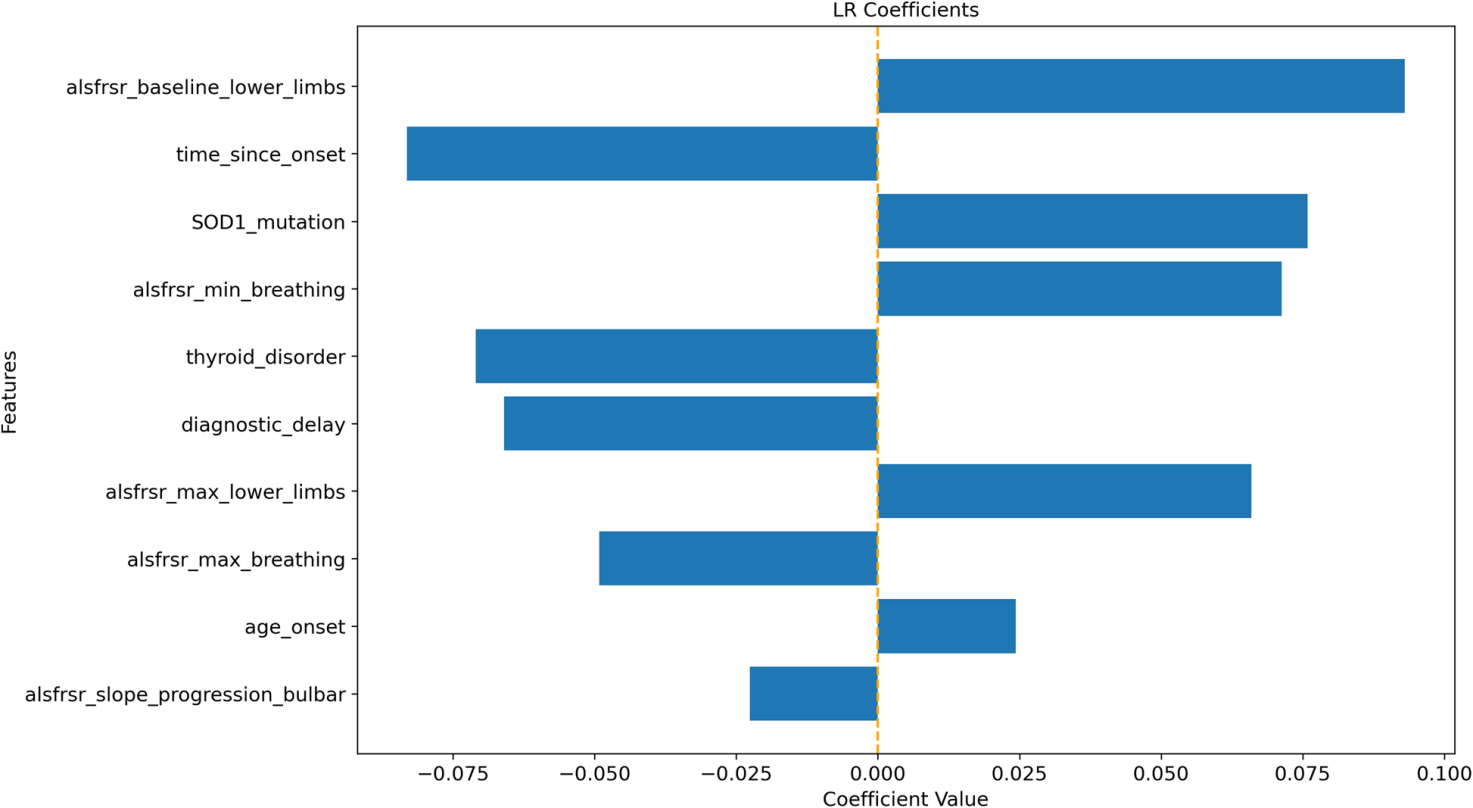
LR coefficients for the 10 most impactful variables of the death prediction model. Positive coefficients (right side of the axis) are associated with factors that may increase the likelihood of death. Negative coefficients are associated with factors that may decrease the death probability

**Fig. 2:**
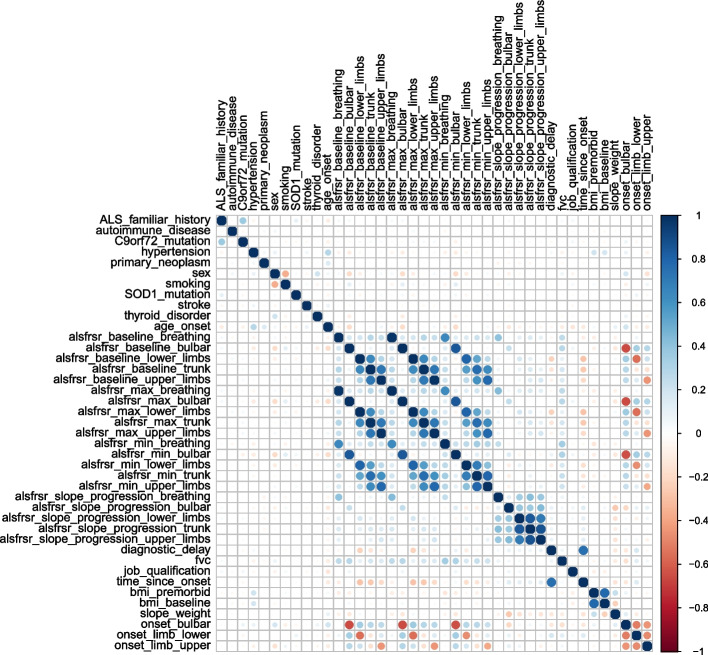
Pearson’s correlation coefficients for all variables combinations. The color blue is associated with a positive correlation meanwhile the color red signals a negative correlation

Figure 1 shows the 10 LR coefficients with the highest absolute value. Notably, factors that emerge as strong predictors of a higher mortality risk are the baseline value of the lower limb ALSFRS-R, SOD1 genetic mutation, the minimum (breathing domain) and maximum (lower limbs domain) values of ALSFRS-R, and age at onset. Instead, time since onset, thyroid disorder, diagnostic delay, maximum ALSFRS-R breathing value, and ALSFRS-R rate of change for the bulbar domain show a negative coefficient. Some of the obtained LR coefficients seem to lead to inconsistent conclusions with respect to what is known from the literature. For example, it is known that patients with high ALSFRS-R scores show better prognoses [27]. However, the LR coefficient associated with the ALSFRS-R baseline value for the lower limbs domain is associated with a positive coefficient signaling an increased risk of death associated with higher values of this variable. The same effect is observed for the minimum values of the ALSFRS-R score for the lower limbs and breathing domain. These observations could be explained by looking at the collinearity heatmap shown in Fig. 2. Here we can definitely see the presence of clusters of collinear variables such as the baseline, minimum and maximum ALSFRS-R values for the various domains, the ALSFRS-R rate of change for the various domains, and the onset types. To properly interpret this result, it is first important to note that the model’s parameters link the variables’ impact on the model’s outcome, not on the real outcome. Therefore, these estimated parameters should not be considered as direct causes of the outcome. Positive/negative associations that are not aligned with the clinical practice knowledge have likely emerged due to numeric compensation effects in a simple linear model such as LR. This effect does change the visualization of the coefficients but does not affect the model prediction, as evidenced by the good performance achieved by the model in the independent test set.

**Fig. 3:**
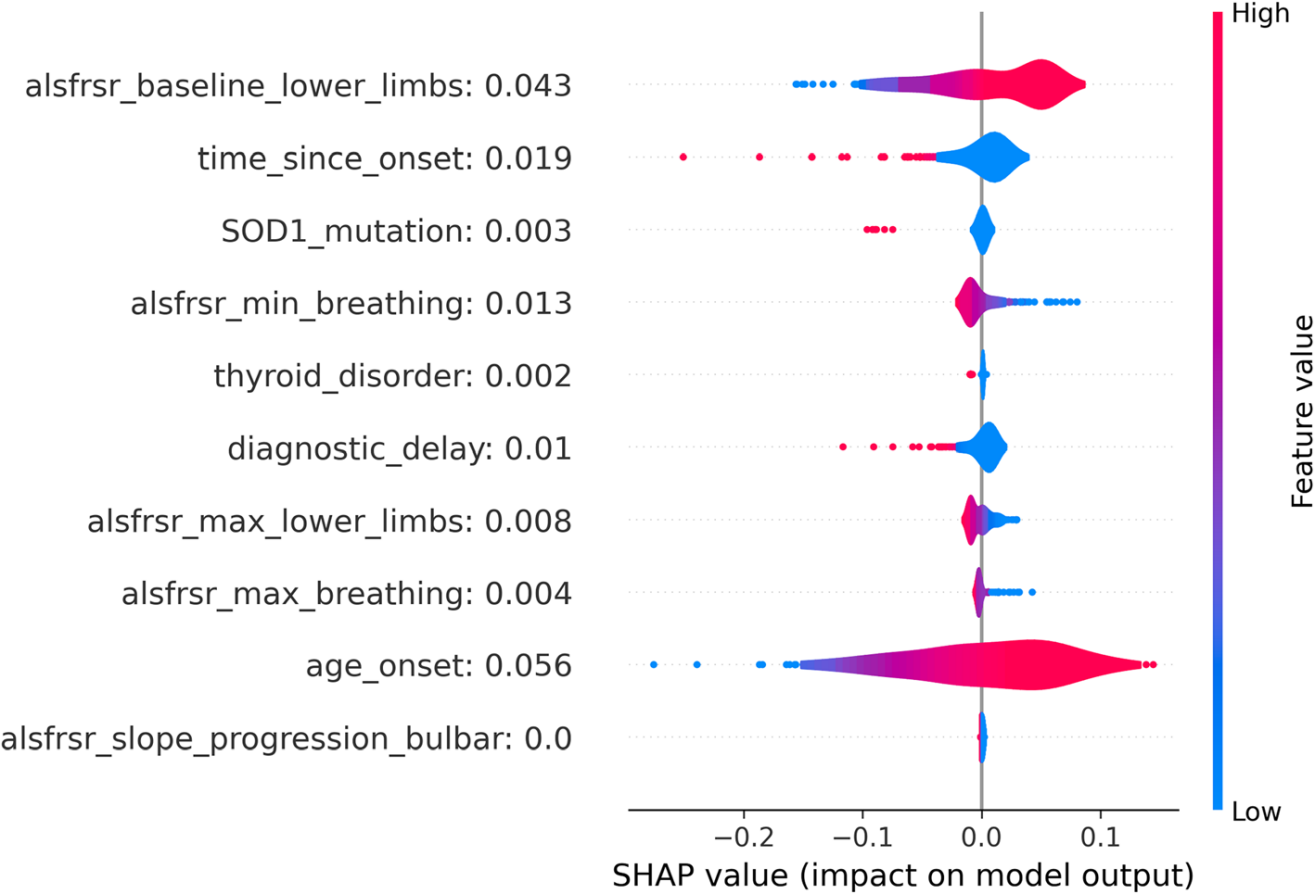
SHAP values distributions for the 10 most impactful variables of the LR death model. Positive SHAP values (right side of the figure) are associated with an increase in death probability. Negative SHAP values are associated with a decrease in death probability. Distributions are color-coded, red portions are associated with high variable values while blue portions with low variable values

To provide an alternative explanation of the LR model, SHAP values were also computed. As shown in Fig. 3 the LR model interpretation obtained through SHAP was closer to the explanation of the parameter effect on the model outcome than the one observed by looking at the coefficients of the model. According to the SHAP interpretation of the LR model, only one variable showed an association not intuitively aligning with clinical knowledge: the ALSFRS-R baseline value for the lower limb domain, whose higher values resulted associated with a higher risk of death. However, this result is related to the characteristics of the analyzed dataset. In fact, all baseline ALSFRS-R scores included in the analysis belonged to ALS patients who received ALS diagnosis according to El Escorial Criteria. Hence, in this population, to have higher ALSFRS-R at baseline in lower limbs means to belong to some of the ALSFRS-R phenotypes in which lower limbs are unaffected at baseline, namely patients with bulbar, respiratory, or upper limbs onsets who, as confirmed by the literature, show a poor disease progression and thus have a higher risk of death [28, 29].

**Fig. 4:**
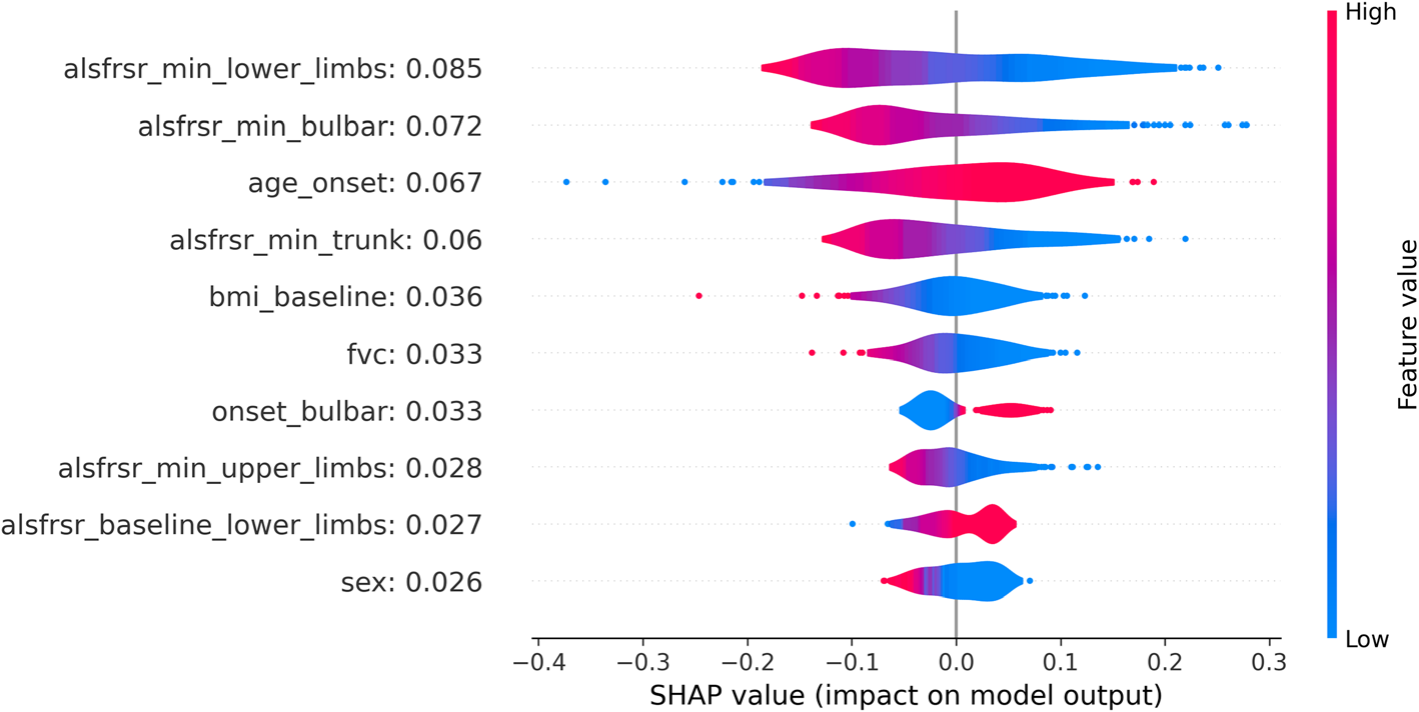
SHAP values distributions for the 10 most impactful variables of the MLP death model. Positive SHAP values (right side of the figure) are associated with an increase in death probability. Negative SHAP values are associated with a decrease in death probability. Distributions are color-coded, red portions are associated with high variable values while blue portions with low variable values

Figure 4 shows the distributions of SHAP values for the top 10 variables with the strongest relationship with the death outcome. These values were obtained when considering the MLP as a methodological approach. The average SHAP of each variable is reported next to the variable name within the figure. According to the information provided by the SHAP values, demographic data, onset characteristics, and ALSFRS score evaluations for different anatomical regions were the most relevant factors influencing the prediction. Higher minimum ALSFRS scores significantly contribute to lower death risk, which is in alignment with clinical expectations. Furthermore, a bulbar onset exhibits a notable influence on the model’s output coherently with the literature describing the bulbar onset as the one leading to the most severe outcomes [30].

Overall, different variables assume greater importance in the two considered approaches when predicting death. In the LR model, variables primarily associated with the progression of ALS scores and those associated with temporal information of the disease (e.g. diagnostic delay and age onset) emerge as critical predictors of the outcome. Instead, within the MLP model, demographic variables (e.g. baseline BMI and sex) together with the ones related to the ALSFRS-R scores, show a higher impact in influencing predictions.

**Fig. 5:**
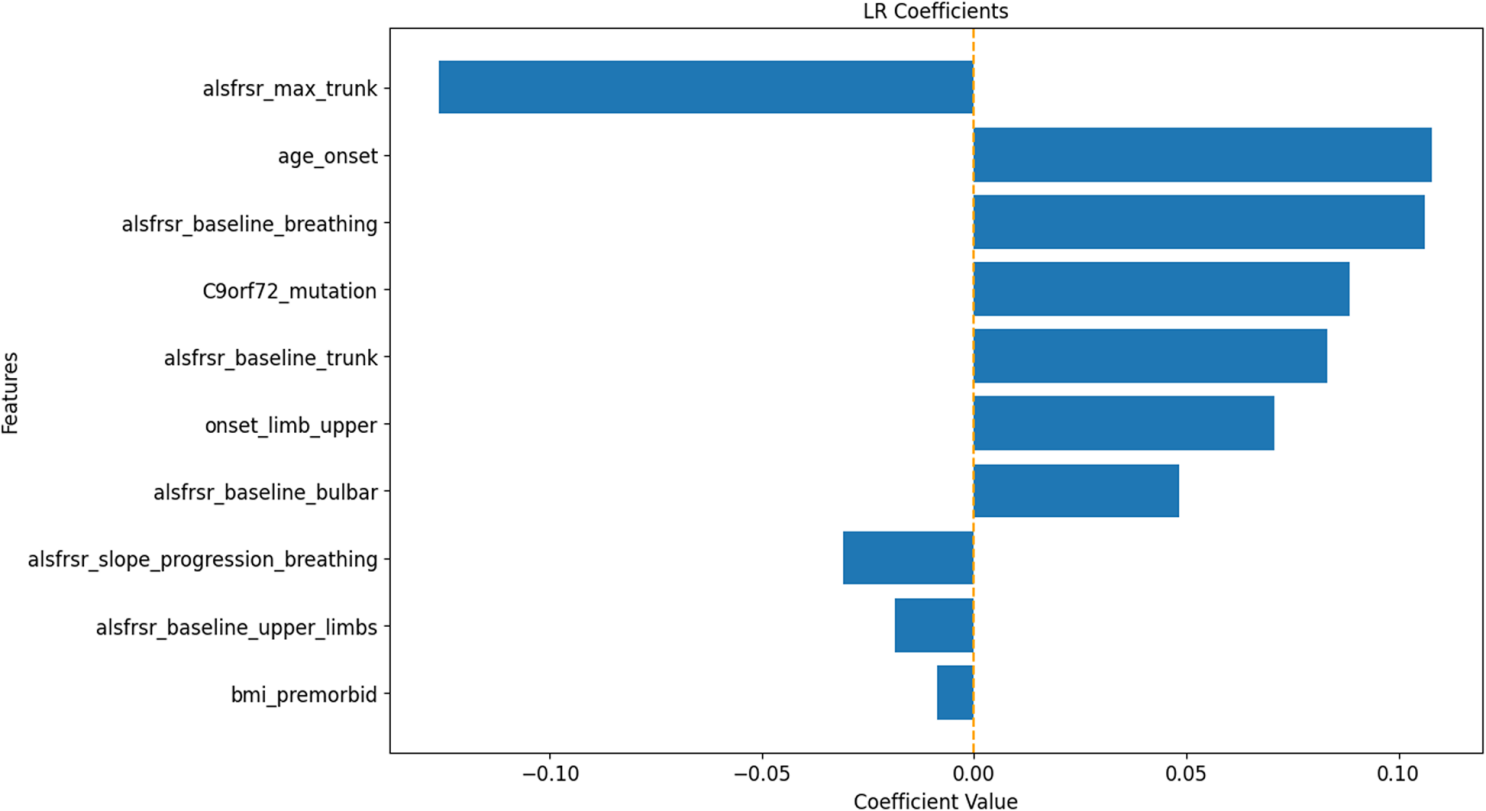
LR coefficients for the 10 most impactful variables of the death or PEG model. Positive coefficients (right side of the axis) are associated with factors that may increase the likelihood of the outcome. Negative coefficients are associated with factors that may decrease the outcome probability

**Fig. 6:**
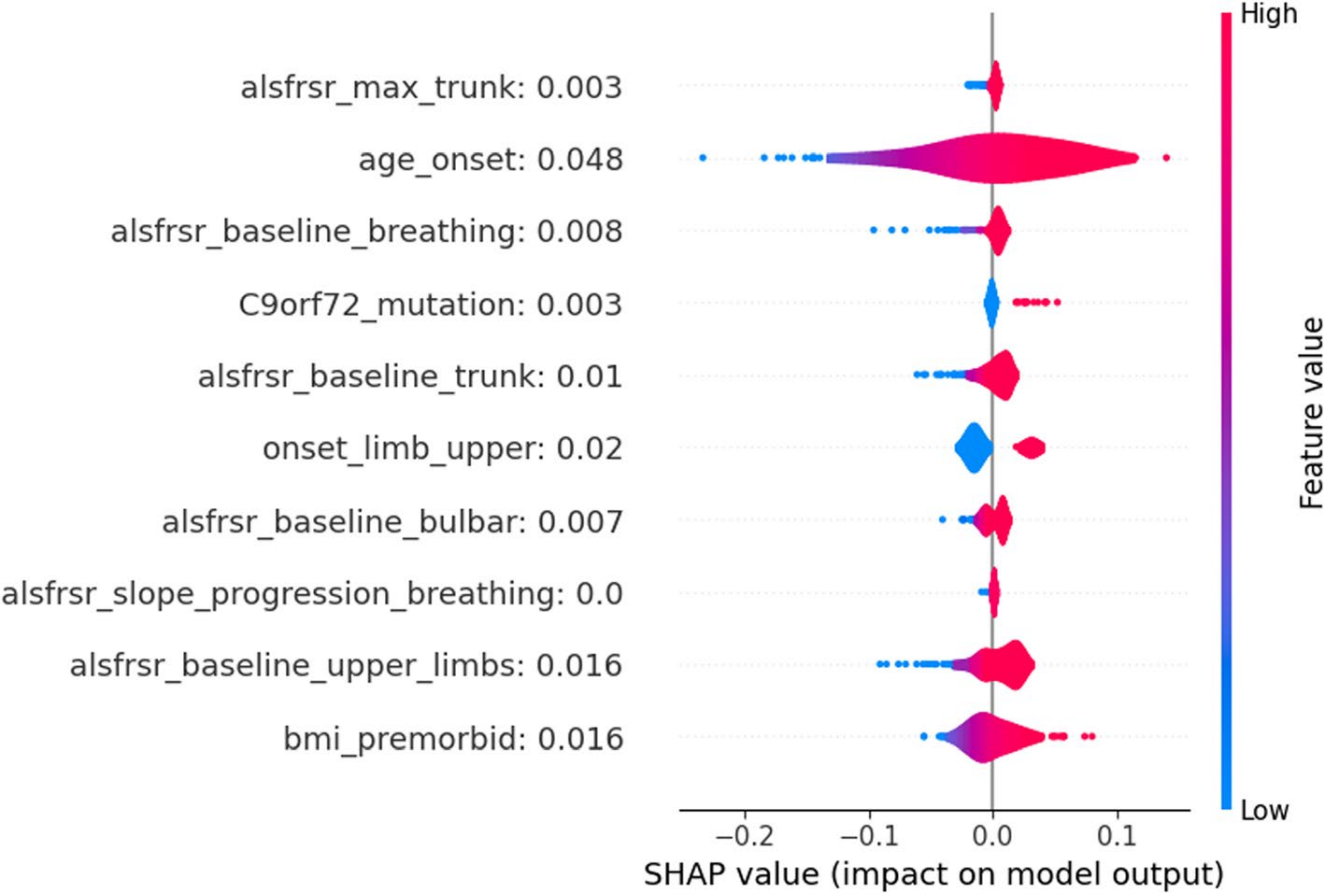
SHAP values distributions for the 10 most impactful variables of the LR death or PEG model. Positive SHAP values (right side of the figure) are associated with an increase in outcome probability. Negative SHAP values are associated with a decrease in outcome probability. Distributions are color-coded, red portions are associated with high variable values while blue portions with low variable values

**Fig. 7:**
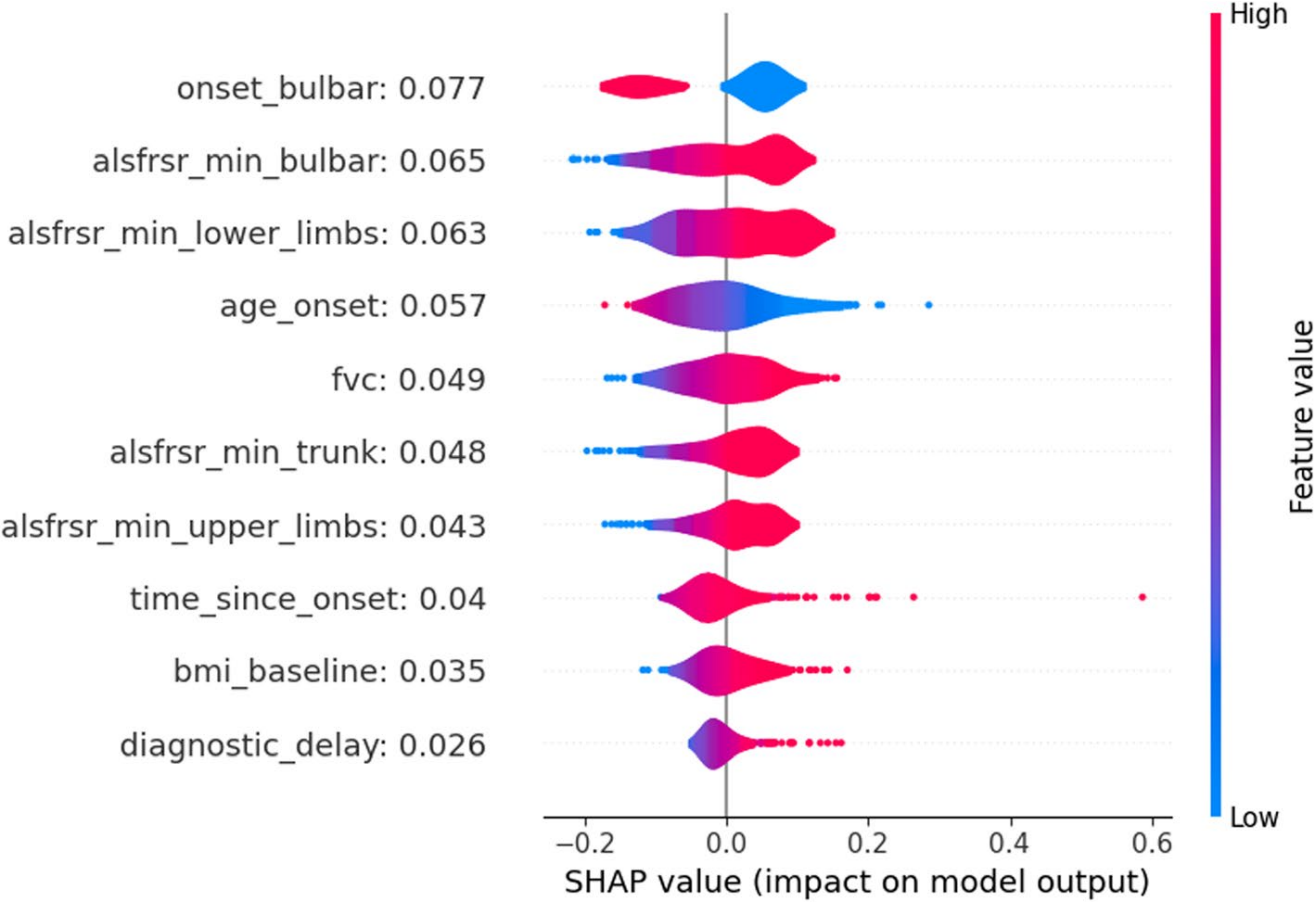
SHAP values distributions for the 10 most impactful variables of the MLP death or PEG model. Positive SHAP values (right side of the figure) are associated with an increase in outcome probability. Negative SHAP values are associated with a decrease in outcome probability. Distributions are color-coded, red portions are associated with high variable values while blue portions with low variable values

**Fig. 8:**
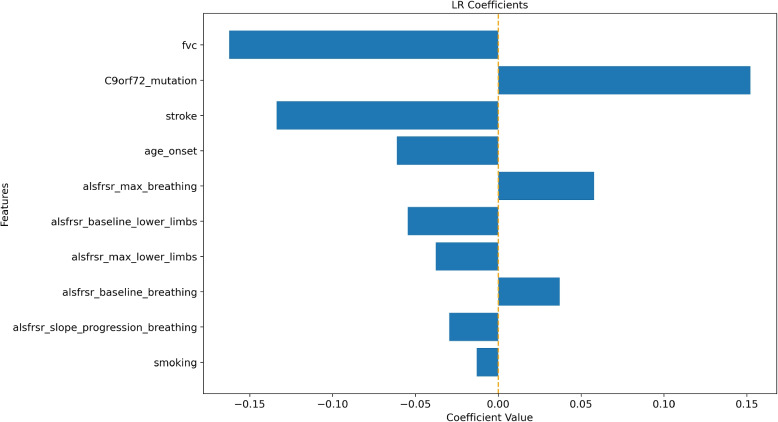
LR coefficients for the 10 most impactful variables of the death or NIV model. Positive coefficients (right side of the axis) are associated with factors that may increase the likelihood of the outcome. Negative coefficients are associated with factors that may decrease the outcome probability

**Fig. 9:**
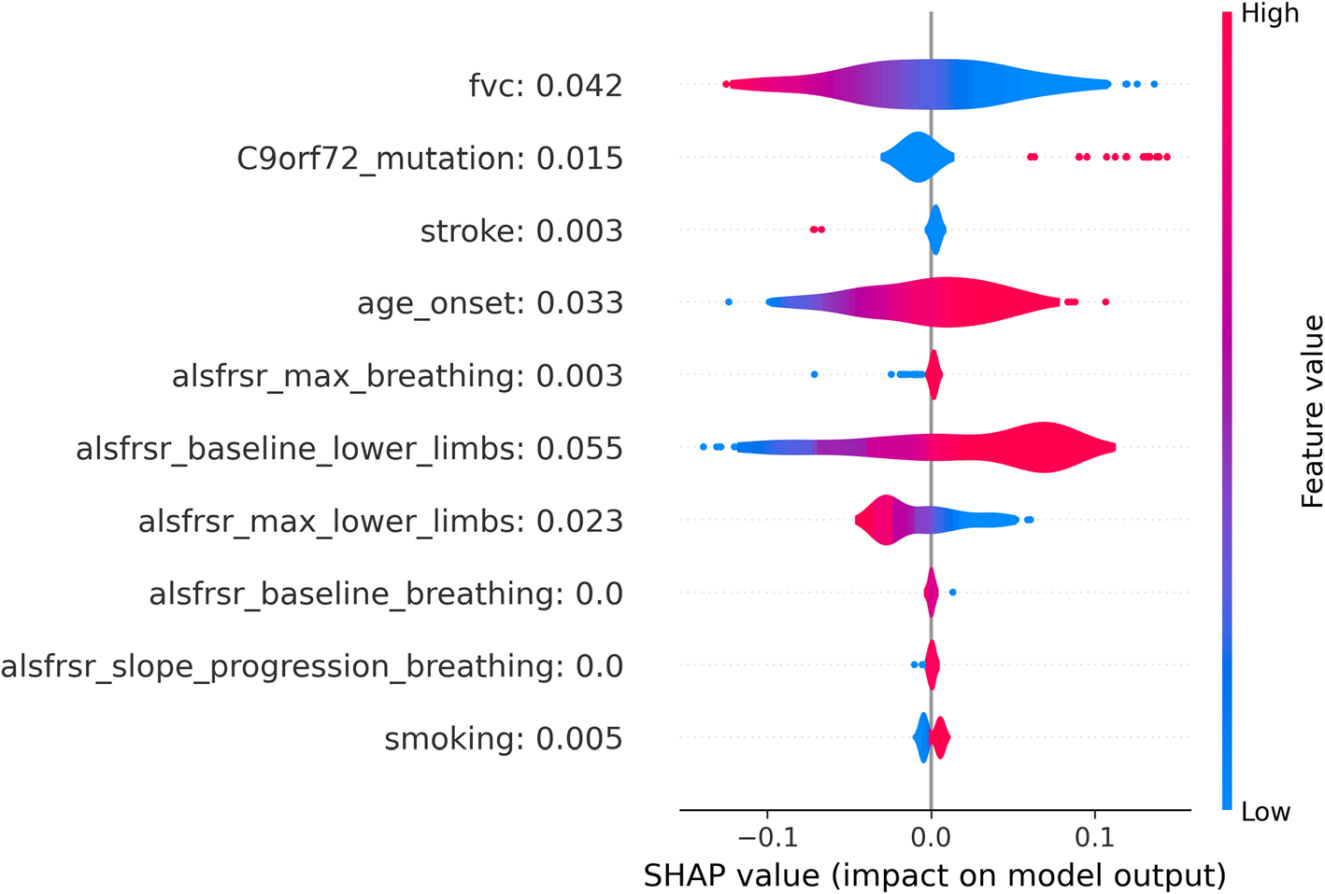
SHAP values distributions for the 10 most impactful variables of the LR death or NIV model. Positive SHAP values (right side of the figure) are associated with an increase in outcome probability. Negative SHAP values are associated with a decrease in outcome probability. Distributions are color-coded, red portions are associated with high variable values while blue portions with low variable values

**Fig. 10:**
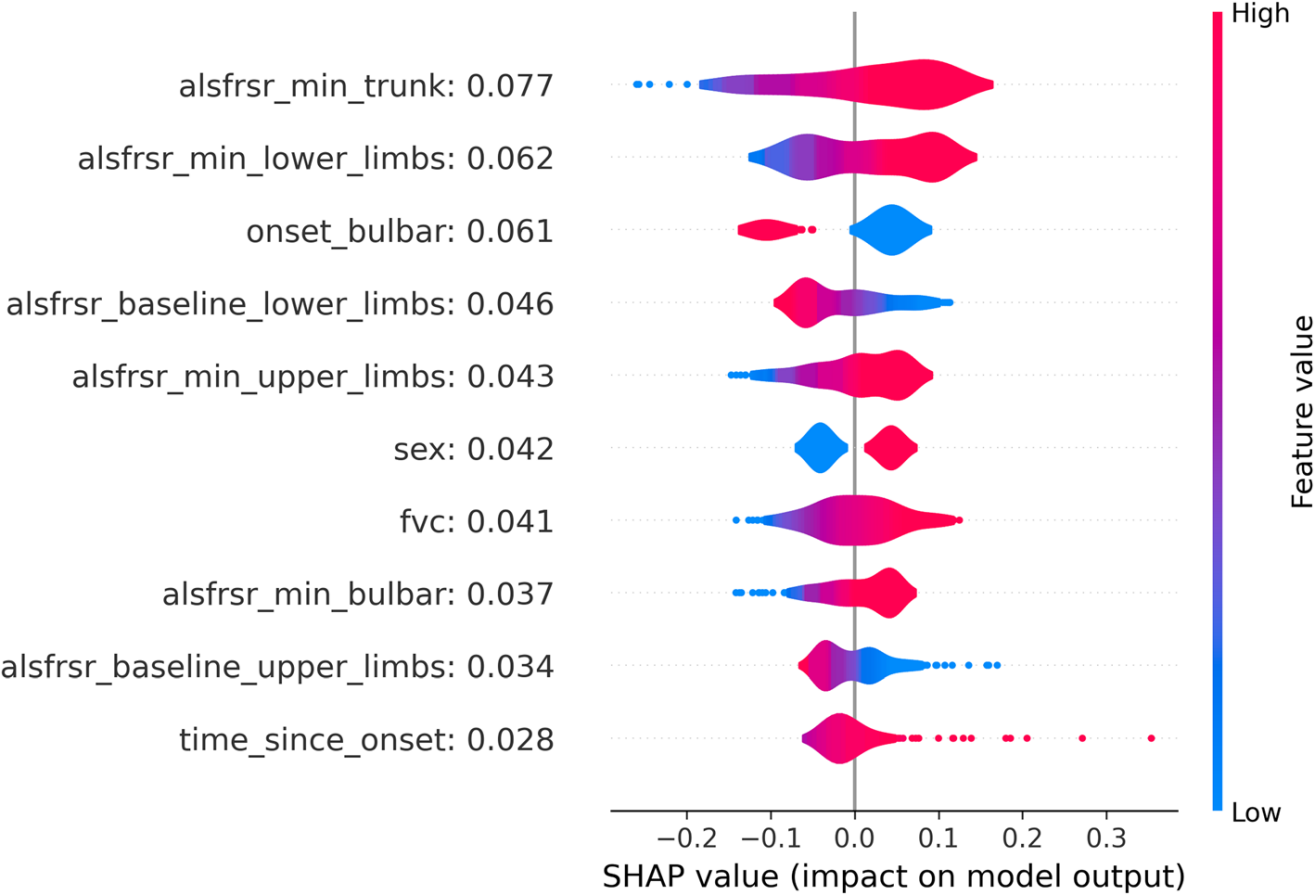
SHAP values distributions for the 10 most impactful variables of the MLP death or NIV model. Positive SHAP values (right side of the figure) are associated with an increase in outcome probability. Negative SHAP values are associated with a decrease in outcome probability. Distributions are color-coded, red portions are associated with high variable values while blue portions with low variable values

Finally, as analyzing incorrect predictions could be useful for debugging purposes, explainability plots for multiclass models can be found below (Figs. 5, 6 and 7 for the death or PEG outcome; Figs. 8, 9 and 10 for the death or NIV outcome).

### Clinical interpretation of explainability results

Insights obtained through SHAP for both the LR and MLP models clearly confirm the effect of prognostic factors that recently emerged in clinical literature. For example, premorbid BMI and BMI at diagnosis have been associated with a slower disease progression [31], confirming the possible benefits of maintenance of body mass through tailored nutritional intervention in ALS [32]. Sex, age at onset, and bulbar onset were confirmed also in our cohorts as associated with an increased death probability [29]. The presence of FVC among the most impactful variables confirmed the role of this pulmonary function test as a marker of the respiratory function [33], while interestingly the ALSFRS-R respiratory items did not result to be significantly associated with survival [34]. All other ALSFRS-R regional subscores (lower limbs, bulbar, trunk, and upper limbs) considered through the minimum score recorded in the 6-month follow-up remain significant as independent predictors of survival, confirming that regional involvement and progression of functional involvement needs to be considered to assess patients’ outcomes [35, 36]. Finally, the use of ALSFRS-R as a multiregional scale or the development of a new multidomain scale is of outstanding importance to better characterize disease severity and progression [37].

## Conclusion

In this work, the data collected in two ALS registries (the Italian PARALS registry and the Lisbon ALS registry) were used to explore the feasibility of developing prognostic models of relevant clinical outcomes of ALS using data collected during routine visits. Specifically, three outcomes were considered in a single and multiclass fashion: namely death (binary outcome), death or PEG (multiclass outcome), and death or NIV (multiclass outcome). Two different modeling approaches were considered: a simple linear approach, i.e., LR, and a more complex non-linear approach, i.e., MLP. This work can be seen as an extension of [6], where the same problem of predicting relevant clinical events for ALS was considered. However, in this previous work, the prediction was tackled using similar data but with a survival analysis perspective, whereas we are now considering a more challenging single and multiclass classification perspective.

On the one hand, models developed to predict death as a binary classification task showed acceptable performance (AUROC $\sim 0.8$ and accuracy = 0.73). These results are in line with those obtained by participants in the intelligent disease progression prediction 2022 challenge (iDPP@CLEF 2022) [38], which used similar data to those available for this study but framed the problem of predicting death in a survival fashion. On the other hand, predicting multiclass outcomes such as death alongside PEG or NIV proved to be more challenging with the available variables.

As the model performance was comparable between the linear and the non-linear techniques, the main driver of predictive performance might be the information that can be extracted from the available data, which includes only variables recorded during routine visits performed at ALS centers. These data have a general nature as they are collected with the aim of giving a broad view of the patient’s disease status rather than performing its full characterization. Model performance could be improved by collecting variables that allow for an in-depth characterization of the disease, such as blood or cerebrospinal fluid tests [39]. However, these measurements are more invasive and their collection process is much more lengthy and expensive. Hence, these data are often not readily available for large amounts of patients as they are typically collected only during clinical trials. This, paired with the fact that clinicians are still studying factors influencing ALS progression and thus many variables are still not known or not measured, is currently a strong limitation towards the development of better-performing predictive models of ALS progression.

In conclusion, our study highlights the potential of AI approaches in complex tasks such as predicting the death of ALS patients using simple data collected during daily clinical practice. However, predicting the occurrence of PEG or NIV alongside death in a multiclass fashion proved to be unfeasible with these data, regardless of the complexity of the chosen methodological approach. Hence, in the future, further studies may focus on the collection or extraction of time-varying and outcome-specific variables as well as the development of more sophisticated methodologies able to consider better temporal information to improve the predictive performance of AI-based approaches.

## Data Availability

The BRAINTEASER ALS data used for this study are available upon request at the URL: https://zenodo.org/records/8083181.

## Abbreviations

ALS: Amyotrophic lateral sclerosis
ALSFRS: ALS functional rating scale
ALSFRS-R: ALS Functional rating scale revised
AI: Artificial intelligence
AUPRC: Area under the precision-recall curve
AUROC: Area under the receiver-operating curve
FVC: Forced vital capacity
LR: Logistic regression
MCC: Matthews correlation coefficient
MLR: Multinomial logistic regression
MLP: Multilayer perception
NIV: Non-invasive ventilation
PARALS: Piemonte and Valle d’Aosta register for amyotrophic lateral sclerosis
PEG: Percutaneous endoscopic gastrostomy
SHAP: SHapley Additive exPlanations
